# Covid 2019 and suicide - a global pandemic: How to prevent?

**DOI:** 10.1192/j.eurpsy.2021.1568

**Published:** 2021-08-13

**Authors:** M.J. Gonçalves, R. André, C. Sereijo, G. Andrade, R. Saraiva, L. Linhares, I. Chendo, M. Abreu

**Affiliations:** 1 Psychiatry, Centro Hospital Lisboa Norte, Lisboa, Portugal; 2 Psiquiatria, Centro Hospitalar Universitario Lisboa Norte, oeiras, Portugal; 3 Psiquiatria, Centro Hospitalar Universitario Lisboa Norte, Lisboa, Portugal

**Keywords:** COVID-19, Suicide prevention, Suicide

## Abstract

**Introduction:**

The mental health effects of Coronavirus2019(COVID-19) outbreak might be profound, including higher suicide rates.This phenomena is likely to become a more pressing concern as the pandemic spreads.While remarkable social distancing interventions have been implemented to reduce the rate of new infections,the potential for adverse outcomes on suicide risk is high, especially among vulnerable populations.

**Objectives:**

The aim is to do a review of the literature of suicide prevention during the COVID-19 outbreak.

**Methods:**

Non-systematic review of the literature with selection of scientific articles published in the last 7 months; by searching the Pubmed databases, the following MeSH terms were used: Suicide prevention; COVID-19

**Results:**

In order to prevent suicide, urgent consideration must be extend beyond general mental health approaches. A wide-ranging interdisciplinary response that recognises how the pandemic might heighten risk is needed. The application of knowledge about effective suicide prevention is the key. Mental health services should develop clear remote assessment and care pathways, and staff training to support new ways of dealing with. Publications on mental health and psychological effects of COVID-19 outbreak provide important information and recommendations for all three levels of suicide prevention: primary, secondary, and tertiary.

**Conclusions:**

The challenge of the COVID-19 outbreak might bring with it an opportunity to advance the field of suicide prevention and, thus, to save lives, which also represent a public health priority. The mental health community, backed by active vigilance and international collaboration, should be prepared and can use this challenging period to advance suicide prevention.

